# Classification of Activity Engagement in Individuals with Severe Physical Disabilities Using Signals of the Peripheral Nervous System

**DOI:** 10.1371/journal.pone.0030373

**Published:** 2012-02-17

**Authors:** Azadeh Kushki, Alexander J. Andrews, Sarah D. Power, Gillian King, Tom Chau

**Affiliations:** 1 Bloorview Research Institute, Holland Bloorview Kids Rehabilitation Hospital, Toronto, Canada; 2 Institute of Biomaterials and Biomedical Engineering, University of Toronto, Toronto, Canada; National Cancer Institute, United States of America

## Abstract

Communication barriers often result in exclusion of children and youth with disabilities from activities and social settings that are essential to their psychosocial development. In particular, difficulties in describing their experiences of activities and social settings hinder our understanding of the factors that promote inclusion and participation of this group of individuals. To address this specific communication challenge, we examined the feasibility of developing a language-free measure of experience in youth with severe physical disabilities. To do this, we used the activity of the peripheral nervous system to detect patterns of psychological arousal associated with activities requiring different patterns of cognitive/affective and interpersonal involvement (activity engagement). We demonstrated that these signals can differentiate among patterns of arousal associated with these activities with high accuracy (two levels: 81%, three levels: 74%). These results demonstrate the potential for development of a real-time, motor- and language-free measure for describing the experiences of children and youth with disabilities.

## Introduction

Individuals with severe and multiple disabilities are often unable to use verbal (speech) or non-verbal (gestures, facial expressions) communication to interact with others. Instead, they rely on alternative and augmentative communication (AAC) methods that complement or replace these communication functions. The typical AAC paradigm requires a user to select messages or codes from a set of possibilities provided by symbol books (e.g., communication boards) or electronic devices (e.g., on-screen keyboards). Symbol selection may be achieved by directly selecting the symbol (e.g., by using a finger or limb or by dwelling with a head mouse or eye tracker) or indirectly with the sequential scanning of symbols coupled with mechanical or bioelectric switch activation. The target population for AAC includes individuals with congenital disabilities (e.g., cerebral palsy and muscular dystrophy) as well as those with acquired conditions (e.g., amyotrophic lateral sclerosis, multiple sclerosis, traumatic brain injury, stroke, and spinal cord injury).

### Alternative and Augmentative Communication: Challenges

The goal of AAC is to enable efficient and effective participation in social interaction and activities [1]. Effective AAC communication, however, is contingent on accurate and timely production of symbols through direct or indirect selection. Direct selection is often a significant challenge for individuals with severe motor disabilities who may have limited and unreliable movements due to hypertonia, hypotonia, and dyskinesia. Moreover, users relying on mechanical switches to control AAC devices face challenges related to positioning and mounting of the switch, inadequacy of activation force (e.g., hypotonia), inability to release a switch after activation (e.g., due to spasticity), false activations due to involuntary movements (e.g., hyperkinetic cerebral palsy), and poor timing [2]. These concerns motivate the development of movement-free control methods for AAC devices. Brain-computer interfaces (BCI) [3] are examples of such methods. These systems generate control signals for external devices by detecting spontaneous or deliberate modulation of brain activity through measurement modalities such as electroencephalography (EEG) [4], [5], near-infrared spectroscopy (NIRS) [6], and ultrasonography [7]. While BCIs may provide the opportunity for selection where none existed, the speed of communication is still dependent on the actual AAC paradigm. For example, the user of an on-screen keyboard is typically required to sequentially scan through a number of letters before the target letter can be chosen [2]. Moreover, fatigue resulting from continuous, conscious control of BCI devices may discourage everyday use by individuals with disabilities [8].

The consequence of the above challenges is that communication using AAC may be fatiguing, slow, prone to errors, and dependent on support from care-givers or communication partners. This limits the quality and quantity of communication and results in asymmetrical interactions that are mainly initiated and dominated by the speaking partner [9]. As such, individuals with complex communication needs typically experience few opportunities for expressive communication [10] and are often marginalized in social settings because of arrangements that do not support their unique needs [11]. Children and youth with disabilities are also often excluded from social and learning activities essential to developing life skills, forming friendships, establishing self-worth and identity, and achieving mental and physical health [12]. Even when physically included, activities of these individuals tend to be characterized by patterns of restricted participation and increased engagement in passive and solitary activities [12].

*Engagement*, defined as the behavioural and emotional quality of involvement [13], reflects a person's active involvement in a task or activity. There is evidence that engagement is a key mediating factor in children's development and positive academic, behavioural, and social functioning [14], [15]. In fact, the degree to which child-care programs promote engagement is considered as an indicator of program quality [16], [17]. Despite this, very little is known about the factors that promote engagement and participation in children with severe disabilities. This is in part due to communication barriers as this group of children has limited verbal and non-verbal means to express their experiences. Moreover, it is suggested that emotional competency, defined as “the process through which children learn to recognize, interpret, use, and respond to emotions”, may develop differently in this group [18]. Collectively, these issues limit the utility of self and care-giver reports in understanding the experience of engagement in children and youth with disabilities. This in turn hinders the development of programs and services that optimally promote engagement and participation in this group of children.

In light of the aforementioned gap, our study investigates the development of movement- and language-free measures that can be used to describe experiences of children and youth with disabilities.

### Measure of Activity Engagement: Signals of the Peripheral Nervous System

It is suggested that engagement is comprised of behavioural (e.g, effort), cognitive (self-regulation), and affective/psychological (e.g., interest) subcomponents [14]. There is evidence to suggest that changes in affective and psychological states can be detected physiologically - specifically using the signals of the peripheral nervous system [19]–[21]. This motivates the investigation of physiological patterns as a language-free means of engagement in children and youth with disabilities.

The peripheral nervous system connects the organs and limbs to the central nervous system (brain and the spinal cord) and is further divided into the autonomic and somatic nervous systems [22]. The autonomic nervous system (ANS) is responsible for regulating visceral functions such as heart rate, respiration, perspiration, and digestion. In the presence of stressors, the arousal of the sympathetic branch of the ANS promotes a number of physiological changes to prepare the body for defensive behaviours. During this stress response, known as the “fight or flight” response, heart and respiration rates increase, pupils dilate, perspiration increases, and blood is diverted away from organs and skin to skeletal muscles and lungs through vasoconstriction (ANS arousal). Once the stress situation is over, the parasympathetic branch of the ANS returns the body to steady state.

Due to the role of the ANS in controlling physiological changes in the presence of external stimuli, the activity of this system is correlated with changes in affective and psychological states [22], [23]. Moreover, the response of the ANS to changes in these states can be detected through measurement of signals reflecting ANS activity (e.g., cardiac and electrodermal activity, skin temperature, and respiration) [24]. In fact, a large body of literature provides evidence that physiological responses resulting from different psychological and affective states can be differentiated [25]. For example, statistically significant differences in indexes of heart rate, electrodermal activity and respiration were reported in response to three psychological states (relaxation, engagement, and stress) [26]. Similarly, Haag et al. [27] automatically classified physiological response patterns corresponding to three levels of arousal (low, medium, high) and two levels of valence (positive and negative) elicited through photo watching in a single individual.

While the above results are promising, the aforementioned studies only involved adults without disabilities. It is unclear whether or not similar results can be obtained with individuals with severe disabilities for three reasons. First, measurement of signals related to ANS activity (e.g., heart rate, electrodermal activity) is severely affected by the presence of motion and pressure artifacts. This poses a challenge to obtaining these signals from individuals with disabilities that result in involuntary movements (e.g., hyperkinetic cerebral palsy). Second, there may be anatomical and physiological differences between individuals with and without disabilities that affect the autonomic nervous system response. Third, some of the individuals in previous studies were professionally trained to modulate their affective states. Such training may not be possible in individuals with disabilities due to differences in cognitive abilities. In this context, encouraging results were reported by a recent study that examined the ANS response of children with severe disabilities during two activities - television watching (low engagement) and therapeutic clowning (high engagement) [28]. Results suggested that the patterns of ANS response may be different between the two activities, lending further evidence to support the development of an ANS-based communication tool. To quantify the activity of the autonomic nervous system, we used blood pulse volume (BVP), electrodermal activity (EDA), respiration, and skin temperature. BVP is related to the changes in the volume of blood in vessels and provides an indirect measure of heart rate which is increased with the arousal of the ANS in response to external stimuli. EDA measures changes in the skin's electrical conductivity due to sweat production by the eccrine glands. Since these glands have sympathetic cholinergic innervations [29], the amount of sweat in the skin, and therefore, skin conductivity, change with ANS activity [30]. Respiration changes are also related to the activity of the ANS as respiration rate is increased during the stress response to accommodate for the increased need for oxygen (note that respiration rate is not completely autonomically controlled as conscious control of respiration rate is also possible). Finally, skin temperature changes occur with changes in sympathetic stimulations of cutaneous microcirculation structures (arteriovenous anastomoses) which lead to vasoconstriction or vasodilation [31].

The second part of the peripheral nervous system is the somatic nervous system (SNS). This system contains nerves that enable voluntary control of the body's skeletal muscles and sensory organs. Interestingly, movement disturbances (loss of typical movement or presence of atypical and involuntary movement) may vary in frequency, intensity and duration with affective states [32] or personal preference [33]. In this light, we hypothesized that the somatic nervous system signals may provide indicators of psychological arousal in individuals with disabilities. We quantified the activity of the somatic system by measuring limb movements.

The above evidence suggests that physiological changes associated with the activity of the peripheral nervous system are related to changes in affective and psychological states. As such, these changes can potentially provide a language-free measure of experiences that are associated with engagement in different activities. As a first step in developing such a measure, we addressed two questions in this study:

1. Can psychological arousal due to activity engagement be detected based on changes in signals of the peripheral nervous system in individuals with severe disabilities?
2. Can signals of the peripheral nervous system differentiate between patterns of engagement resulting from different activities in individuals with severe disabilities?

## Methods

### Ethics Statement

The Bloorview Research Institute research ethics board approved the study and all participants provided informed consent. A special process was approved by the ethics board to obtain consent from participants who were nonverbal or unable to provide written consent due to physical abilities. The process was as follows. Prior to the study, potential participants and their parents received an information package to ensure they have an opportunity to fully review the study information and consent forms. During the study visit, a research assistant (RA) provided participants and their parents with a detailed overview of the study. For youth with little or no functional speech, a pictorial overview of the study components was provided along with verbal explanations. Prior to completion of the consent forms the RA asked a series of questions to determine the youth's capacity to consent to participate in the study. If the youth's responses to the questions demonstrated the ability to understand the purpose of the study and their rights regarding participation in the study then the RA determined that consent could be provided, otherwise the youth were excluded from the study. Following determination of capacity to consent, the Picture Communication Symbols (PCS) visual consent framework was used to obtain consent. This involved arrangement of communication symbols on a board. The participant either indicated “yes” using their communication aid or using their gestural “yes sign” (e.g. head nod, smile). If the participant was able to sign or had a signature stamp or was able to mark an “X” they also provide written consent. If not, the RA noted that verbal consent was obtained on the written consent form and witness it.

### Participants

We recruited nine individuals with severe physical disabilities (18.1±2.2 years old, 7 female) through Holland Bloorview Kids Rehabilitation Hospital in Toronto, Canada. All participants had cognitive functioning and language comprehension abilities of at least a grade three level.

The group of participants consisted of individuals with cerebral palsy, muscular dystrophy, and an undiagnosed neuro-muscular degenerative disorder. Of the nine participants, seven relied on AAC devices for communication and two had complex continuing care needs (CCC) (*e.g.*, mechanical ventilation, parenteral nutrition, oxygen therapy, tracheotomy). Participant 2 also had a cardio-pulmonary condition. All participants used wheelchairs for mobility.

### Apparatus

**Figure 1:**
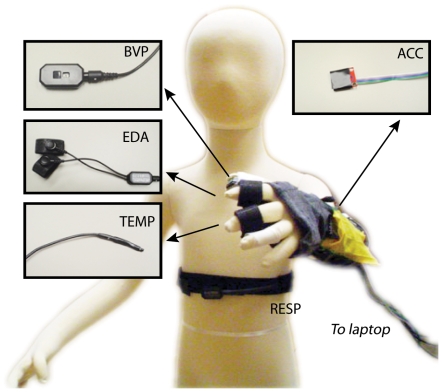
Typical sensor setup. BVP: Blood Volume Pulse, EDA: Electrodermal Activity, Temp: Temperature, Resp: Respiration.

We measured blood volume pulse (BVP), electrodermal activity (EDA), skin temperature and respiration signals using FDA-approved sensors and encoders (Flexcomp Infiniti, Thought Technology Ltd.). BVP, EDA, and skin temperature sensors were attached to the participants using breathable tape and/or velcro straps to either the hands or the feet of the participants, depending on signal quality and the participant's comfort. The sensors and locations are listed below and discussed in detail in [34]. Figure 1 depicts the typical attachment of sensors to the hand.

- To measure BVP, a single photoplethysmography [35] sensor was used. This sensor was secured to the palmar or plantar surface of the distal phalanges of the first digits of the hand or the foot.
- EDA was measured as skin conductance obtained using a pair of 10 mm diameter dry Ag-AgCl electrodes. These sensors were secured to the palmar surface of the second or proximal phalanges of the second and third digits of the non-dominant hand or to the plantar surface of the distal phalanx of the second and third digits of the non-dominant foot. Prior to attaching the sensors, we ensured that the skin was clean and free of visible lesions.
- To measure skin temperature, a thermistor was fastened to the palmar surface of the distal phalanx of the fifth digit of the non-dominant hand or to the plantar surface of the distal phalanx of the fifth digit of the foot.
- Respiration was measured using a piezoelectric belt positioned around the thoracic cavity.
- Limb movement was measured using a tri-axial accelerometer mounted on the same limb as the other sensors.

All signals were sampled at a frequency of 256 Hz and recorded to a laptop computer for subsequent off-line analysis.

### Task

The participants performed three different activities:

- Activity 1 (no engagement required): participants sat in their chair or wheelchair and were not instructed to perform any particular task.
- Activity 2 (passive engagement required): participants passively watched pictures of items they liked or disliked. Picture watching is known to be an effective means to induce emotions [27] and standard affective picture databases have been developed specifically for this purpose (for example, the International Affective Picture System (IAPS)[36]). The IAPS set, however, is validated using participants without disabilities [37]. Considering this and the uniqueness of the individuals in our participant group, we asked the participants to provide prior to the study, a list of 10 items they strongly liked and 10 items they strongly disliked (e.g., objects, feelings, activities). A research assistant then obtained pictures of each item which were used as stimuli. During the activity, participants were instructed to looked at the pictures and reflect on their feelings about the displayed items.
- Activity 3 (active engagement required): participants interacted with a research assistant to communicate their feelings towards the pictures they viewed. The participants indicated how they felt while watching the picture by selecting a grade of agreement with the statement “I felt good when I saw the picture”. The choices for the grades of agreement were “I really agree”, “I agree”, “I don't agree or disagree”, “I disagree”, “I really disagree” [38]. Participants responded with a yes or no to the options read aloud by the research assistant using their typical means of communication (e.g., nod, smile, or vocalizations) or by vocalizing their choice. Any pictures associated with a neutral rating (“I don't agree or disagree”) were excluded from the analysis.

### Protocol

**Figure 2:**
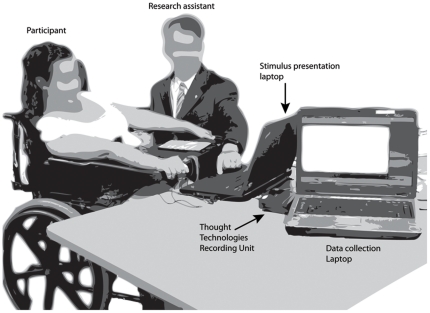
Typical experimental setup. Participants sat in a chair or wheelchair, facing a laptop computer.

**Figure 3:**
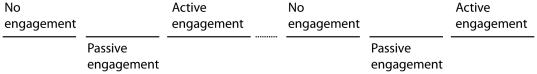
Experimental protocol. Participants alternated between intervals of no engagement (blank screen), passive engagement, and active engagement.

During data collection, participants sat in either a chair or wheelchair, facing a laptop computer (see Figure 2). A five-minute baseline recording was collected to allow for thermal acclimation and relaxation. This was followed by a 20–25 minute period (Figure 3) when participants alternated between 20-second intervals of no engagement (blank screen) and passive engagement (watching a picture on the laptop computer). After each picture, participants rated their experience of the picture as described above in the description of Activity 3. The average length of the rating period was 20±12 seconds.

### Analysis

#### Preprocessing

The data from each session were preprocessed to remove movement, pressure, and physiological artifacts. Table 1 shows the filters used for preprocessing each measured signal. For BVP, a bandpass filter retained frequency content corresponding to 40–200 beats per minute based on typical heart rate limits. As changes in EDA typically happen on the scale of several seconds, a low-pass filter was employed to reduce movement artifacts and high frequency noise above 5 Hz from this signal [39]. The respiration signal was bandpass filtered preserving frequency content in the range of 10–120 breaths per minute. For the accelerometer, the signal along each axis was adjusted for the effect of gravity by subtracting the mean. We then extracted the norm of centered axial signal (square root of the sum of squared values along the three axes) for further processing.

**Table 1:** Filters used to preprocess the data.

Signal	Filter type
BVP	3rd order Butterworth bandpass filter (0.67–3.33 Hz)
EDA	2nd order Butterworth lowpass filter (5 Hz)
Temperature	No filtering used
Respiration	3rd order Butterworth bandpass filter (0.17–2.00 Hz)
Acceleration	Mean adjusted

After filtering, linear trends over the entire session were removed from the data to mitigate the effects of thermal regulation (for example, general increases in temperature or thermoregulatory perspiration) and changes in posture.

For each participant, the data were segmented to extract the intervals corresponding to periods of no engagement, passive engagement, and active engagement (20 intervals for each activity type).

#### Feature Extraction

Four features were extracted from each of the physiological signals from the no engagement, passive engagement, and active engagement intervals:

- Mean and standard deviation of signal values over the interval;
- Slope: this feature was extracted as the slope of the line-of-best fit over the interval;
- First order difference (FOD): this feature is computed as the mean of the absolute values of the first differences of the signal standardized by its mean and variance over the interval [40].

For the BVP signal, the mean, standard deviation, and slope were obtained from the instantaneous heart-rate values extracted using a custom shape-matching algorithm. Similarly, the aforementioned features were computed from the instantaneous respiration rate obtained through peak detection. For the accelerometer, feature extraction was performed on the norm of the the mean-corrected signal.

With the goal of eventual real-time operation, the features used for activity classification were computed as the change in each of the features between consecutive intervals. For example, the features used for the active engagement interval were obtained as the feature differences between the active engagement period and the preceding passive engagement interval.

#### Classification and Feature Selection

We employed linear discriminant analysis (LDA) [41] to automatically classify the activity in which the participant was engaged using the measured set of autonomic and somatic nervous system signals. LDA projects signal patterns into a lower dimensional space where the patterns corresponding to each class are maximally separated. We examined three, two-class classification problems (no engagement versus passive engagement, no engagement versus active engagement, and passive versus active engagement) as well as the three-class problem (no engagement versus passive versus active engagement). Classification accuracy was calculated as the percentage of correctly classified samples obtained from 100 iterations of 5-fold cross-validation.

Previous literature [28], [40] suggests that the ANS response to external stimuli may be more pronounced in a subset of the physiological signals and that this subset may be different for each individual. In this light, we employed the Sequential Feature Forward Selection with the Fisher criterion [41] to determine the most separable features for activity classification for each individual. This criterion is defined as:
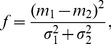
(1)where 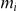 and 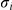 are the mean and variance of feature values for class 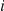. For the three-class problem, we used the sum of the Fisher criterion values computed for each pair of classes.

## Results

Classification accuracies for the four classification problems are reported for each participant in Table 2 (4 and 9 features were used for the two-class and three-class problems, respectively). These accuracies significantly exceeded chance for all classification problems and all participants with three exceptions: participants 1, 3, and 7 (note that upper confidence limits of chance results for 2 and 3 class problems with 20 trials per class are 65 and 45, respectively [42]).

**Table 2:** Classification accuracy results (upper confidence limit of chance results for 2 and 3 class problems with 20 trials per class are 65 and 45, respectively [42]).

Participant	No Eng./Passive	No Eng./Active	Passive/Active	No Eng./Passive/Active
**1**	†64±5	†68±5	†65±6	51±5
**2**	97±2	97±1	88±3	94±2
**3**	†52±7	72±5	72±5	56±5
**4**	90±4	89±3	87±3	81±3
**5**	90±2	97±2	96±3	94±2
**6**	87±3	90±3	79±4	75±4
**7**	†50±6	†67±6	†61±5	†45±5
**8**	90±2	84±3	81±4	77±4
**9**	88±4	100±1	92±2	90±4
**Average**	**79**±**18**	**85**±**13**	**80**±**12**	**74**±**19**

† classification accuracy not significantly different from chance (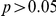). Note - Eng.: Engagement.

**Figure 4:**
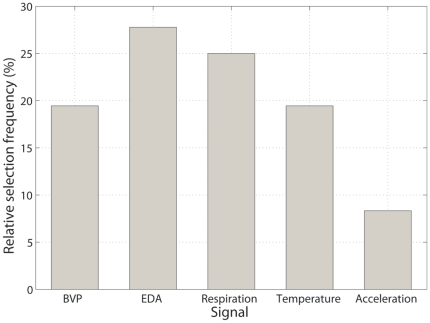
Relative feature selection frequency. EDA features were most commonly picked, followed by respiration and BVP features. The least commonly chosen features were those of limb acceleration.

Table 3 shows the effect of the number of features on classification accuracy for each of the three-class classification problem. As seen, classification accuracy is not highly sensitive to the number of features used for classification. The top four features selected for three-class classification are shown in Table 4 for each participant. These results show that signals of the autonomic and somatic nervous systems were both used for activity classification. Figure 4 shows the relative frequency of each signal appearing in the top four selected features. Based on this figure, EDA features were most commonly picked, followed by respiration and BVP features. The least commonly chosen features were those of limb acceleration.

**Table 3:** Effect of the number of features on classification accuracy (three-class problem).

Part.	1 ft.	2 ft.	3 ft.	4 ft.	5 ft.	6 ft.	7 ft.	8 ft.	9 ft.
**1**	52±5	51±4	54±5	53±5	52±5	51±5	51±5	52±5	52±5
**2**	90±1	88±3	89±3	89±3	91±2	92±3	93±2	94±2	94±2
**3**	66±2	54±5	52±5	51±5	51±5	52±5	53±4	54±5	56±4
**4**	66±5	69±5	72±4	74±4	77±3	78±4	79±3	80±4	81±3
**5**	54±5	73±4	83±4	85±3	85±2	87±2	91±3	93±2	95±2
**6**	53±5	72±5	78±4	74±4	73±4	73±4	74±4	74±4	74±4
**7**	39±6	43±5	48±5	46±5	46±5	46±5	45±4	45±5	45±5
**8**	68±2	70±4	74±4	77±4	80±4	81±4	80±4	79±4	78±4
**9**	56±4	69±4	79±3	84±2	86±3	89±3	90±3	90±3	90±4
**Avg.**	60±14	66±14	70±15	70±16	71±17	72±18	73±18	73±19	74±18

Note - Avg. ft.: features; Part.: Participant #.

**Table 4:** Features most commonly selected for classification based on the Fisher criterion (most frequently selected appears on the left).

Participant	Feature			
**1**	Resp. (FOD)	Resp. (SD)	Accel. (SD)	Resp. (mean)
**2**	EDA (slope)	EDA (FOD)	Temp. (FOD)	EDA (SD)
**3**	Accel. (FOD)	Resp. (mean)	Resp. (SD)	HR (mean)
**4**	Resp. (SD)	EDA (SD)	Resp (mean)	BVP (FOD)
**5**	Accel. (slope)	Temp (slope)	BVP (FOD)	EDA (slope)
**6**	EDA (mean)	Resp. (FOD)	Temp (FOD)	Temp (SD)
**7**	EDA (SD)	Temp (FOD)	Resp. (FOD)	Temp (slope)
**8**	HR (mean)	HR (slope)	EDA (slope)	HR (SD)
**9**	HR (mean)	Temp (FOD)	EDA (slope)	EDA (mean)

Accel.: Limb acceleration, BVP: blood volume pulse, EDA: electrodermal activity, HR: heart rate, FOD: first order difference, Resp: respiration, Temp: skin temperature.

To verify that the classification accuracies were not due to differences in amount of vocalization or physical effort among tasks, we further performed the classification using only the accelerometer and respiration features. Classification accuracy based on all features was significantly higher than that based on respiration or accelerometer features for all participants (Wilcoxon rank-sum, 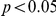), except for participants 1, 3, and 7. Using video records of each session, two evaluators investigated whether or not there were significant differences among patterns of participant movement for the three tasks. Such a pattern was only suspected for participant 5, who used gestures to communicate through an AAC device.

## Discussion

### Peripheral Nervous System Signals as a Means of Communication

We examined the feasibility of using signals of the peripheral nervous system for developing a language-free measure of experience in youth with severe disabilities. We demonstrated that these signals can be used to automatically differentiate between activities requiring no participant engagement, passive engagement, and active engagement. In particular, we showed that both passive and active engagement activities could be reliably differentiated from the no engagement state. This suggests that psychological arousal associated with activity engagement can be detected in this population despite the presence of anatomical and physiological conditions that may potentially affect signal lability (e.g., mechanical ventilation) and signal acquisition. Moreover, our results indicated that peripheral nervous system signal patterns associated with passive and active engagement were differentiable with higher than 80% accuracy. Overall, our results suggest that signals of the peripheral nervous system provide a promising avenue for the description of experiences in individuals with disabilities. An experience indicator based on peripheral nervous system responses is speech-free and does not rely on language proficiency or voluntary movement control.

Three of the nine participants had poor classification accuracies for differentiation of of the combinations of the three activities (participants 1, 3, and 7). One reason for this may be that these participants had excessive involuntary movements that resulted in significant artifacts in the measured signals.

### Feature Selection

In this study, we used signals of the autonomic and somatic nervous systems to quantify the activity of the nervous system. As seen in Table 4, signals of both branches of the peripheral nervous system were chosen for classification, though the autonomic nervous systems features were more prevalent. Interestingly, somatic nervous system features were only chosen for participants with spasticity. This may suggest some specificity in the selected features with respect to movement characteristics, though further exploration of this issue was not possible in our study due to the small sample size.

Among the signals of the autonomic nervous system, electrodermal activity and respiration were most frequently selected, followed by blood volume pulse and temperature when all participants were considered. This pattern is consistent with that reported in [26] who investigated the response of the ANS to activities inducing states of relaxation, engagement, and stress in participants without disabilities. The fact that some signals were more reflective of psychological arousal than others may be in part due to the differences in the sensitivity of the underlying neural mechanisms to arousal. In particular, changes in electrodermal activity, respiration, and fingertip temperature are primarily attributed to sympathetic activity [24], [31], though changes in fingertip temperature often occur on a much slower scale than the other two signals (for example, he response latency of electrodermal activity is 1–3 seconds [29] while that of fingertip temperature is 15 seconds [31]). In contrast, cardiac activity is affected by the antagonistic interaction of the sympathetic and parasympathetic systems. Given the physiological uniqueness of our sample, further investigations are needed to describe the contribution of each branch of the nervous system to the differentiability of response patterns.

When we considered participants individually, we noted that the subset of features providing the best classification accuracy was not always consistent with the general pattern discussed in the previous paragraph. Determination of this optimal subset is critical for our target population as each individual may have unique physiology. In this study, we employed a feature selection method based on the Fisher criterion to automatically determine the optimal feature subset for classification. This type of feature selection method provides automatic personalization essential to account for inter-individual physiological differences. For example, two of our participants (2 and 9) relied on mechanical ventilation. As seen from Table 4, respiration features were not chosen by the automatic feature selection method for use in classification for these participants. Moreover, features related to cardiovascular activity were not selected for participant 2 who had a cardio-pulmonary condition.

### Limitations

Our results demonstrate the feasibility of classifying activity engagement that is potentially associated with different patterns of psychological arousal related to engagement. We did not, however, examine whether or not the valence associated with the emotional experience of the activity (positive or negative) can also be classified using signals of the peripheral nervous system. While previous studies have reported success in participants with disabilities, the complexity of inducing pure emotions and obtaining self-reports in the target population prevented us from directly replicating those experiments.

This study examined somatic nervous system signals obtained from one limb only. This was done to minimize any confounding effects resulting from voluntary movements used to produce vocalizations and/or to control AAC devices (participants typically used one limb and/or their head for this purpose). An interesting future direction would be to examine whether or not movement data collected from multiple bodily sites can improve the discrimination power of the signals of the somatic nervous system.

Another limitation of this study was that we assumed prior probabilities for the three states. In reality, however, states of engagement are expected to occur less frequently than states of no engagement in the target population. Future investigation of the effect of unequal probabilities on classification accuracy is strongly warranted.

Finally, the order of task presentation was not randomized in this study to ensure a natural progression (rest, picture watching, and evaluation). Because physiological data closer in time tend to be more correlated than those further apart in time, the effect of task order on classification accuracy should be further investigated.
